# Effect of Corona Discharge Treatment on the Adhesion of Polyetheretherketone Using Resin Cement

**DOI:** 10.3390/dj14090609

**Published:** 2026-09-20

**Authors:** Saho Tsukada, Takahiro Wada, Kei Ushijima, Noriko Hiraishi, Masaomi Ikeda, Yasushi Shimada, Mao Hori, Akira Matsumoto, Motohiro Uo

**Affiliations:** 1 Department of Advanced Biomaterials, Graduate School of Medical and Dental Sciences, Institute of Science Tokyo, 1-5-45, Yushima, Bunkyo-ku, Tokyo 113-8549, Japan; tsukada.saho@tmd.ac.jp (S.T.); wada.abm@tmd.ac.jp (T.W.); 2 Department of Cariology and Operative Dentistry, Graduate School of Medical and Dental Sciences, Institute of Science Tokyo, 1-5-45, Yushima, Bunkyo-ku, Tokyo 113-8549, Japanhiraope@tmd.ac.jp (N.H.); shimada.ope@tmd.ac.jp (Y.S.); 3 Department of Oral Biomedical Engineering, Graduate School of Medical and Dental Sciences, Institute of Science Tokyo, 1-5-45, Yushima, Bunkyo-ku, Tokyo 113-8549, Japan; ikedsdt@tmd.ac.jp; 4 Department of Organic Biomaterials, Laboratory for Biomaterials and Bioengineering, Institute of Science Tokyo, 2-3-10 Kanda Surugadai, Chiyoda-ku, Tokyo 101-0062, Japan; hori.mao@tmd.ac.jp (M.H.); matsumoto.bsr@tmd.ac.jp (A.M.); 5 Department of Materials Engineering, Graduate School of Engineering, The University of Tokyo, 7-3-1 Hongo, Bunkyo-ku, Tokyo 113-8656, Japan; 6 Innovation Center of NanoMedicine (iCONM), Kawasaki Institute of Industrial Promotion, 3-25-14 Tonomachi, Kawasaki-ku, Kawasaki 210-0821, Japan

**Keywords:** CAD/CAM, PEEK, surface treatment, corona discharge, tensile bond strength

## Abstract

**Background/Objectives**: Although polyetheretherketone (PEEK) has been widely used for its excellent properties, its chemical inertness remains an ongoing challenge. This study aims to evaluate the effects of corona discharge (CD) treatment on the adhesion of dental PEEK to resin cement. **Methods**: Mirror-polished (MP) or sandblasted (SB) PEEK specimens were divided into CD-treated [CD(+)] and -untreated [CD(−)] groups. Following primer application, the specimens were bonded with resin cement and subjected to either 0 (TC0) or 5000 (TC5000) thermal cycles. The tensile bond strength (TBS) was measured and statistically analyzed using one-way ANOVA and *t*-tests (α = 0.05). Failure modes were analyzed and classified as adhesive, mixed, or cohesive. The surface roughness and contact angle were also evaluated. **Results**: CD treatment increased the surface roughness of the MP specimens while slightly decreasing that of the SB specimens. The wettability of both surfaces was significantly improved after CD treatment. However, no significant differences in TBS between the CD(−) and CD(+) groups were observed for either the MP or SB specimens. No significant differences in TBS were observed under different TC durations, regardless of CD treatment. **Conclusions**: Although CD treatment significantly improved the surface roughness and wettability of dental PEEK, it did not enhance the TBS of either the MP or SB surfaces.

## 1. Introduction

With the advancement of computer-aided design/computer-aided manufacturing (CAD/CAM) technology, new metal-free restorative materials have become available, with polymer-based composite materials being among the most popular options. For instance, polyetheretherketone (PEEK), a semi-crystalline thermoplastic composed of aromatic rings connected by ether and ketone linkages [1], was originally developed as an industrial material. Recently, PEEK has also drawn attention in the field of dentistry due to its excellent mechanical properties, heat resistance, chemical stability, and biocompatibility, thereby expanding its clinical application [1,2,3]. However, its chemical inertness and lack of surface functional groups [4] result in weaker bonding compared with conventional adhesive systems [5,6]. Consequently, establishing reliable methods to improve the bond strength of PEEK is essential.

Various approaches have been explored for PEEK surface modification, including surface treatments such as sulfuric acid etching [7,8], laser treatment [9], low-pressure plasma [7,8,10], and atmospheric-pressure plasma [10,11,12]. In the field of dentistry, the bond strength between PEEK and resin-based materials has been investigated under various conditions, evaluating the effects of different luting agents, bonding systems, filler contents, surface treatments, and their combinations to enhance the adhesion.

Surface treatments can be classified into mechanical, chemical, and physicochemical approaches. For example, airborne-particle abrasion with aluminum oxide (sandblasting) is a well-established mechanical surface treatment option for PEEK [5,13]. Other approaches, including chemical etching [14,15,16], ultraviolet (UV)/ozone treatment [17], laser treatment [15,18], low-pressure plasma [19,20], atmospheric-pressure plasma [15,21], and a combination of sandblasting and plasma treatments [18,22,23,24,25,26] have also been reported. Furthermore, the combination of sandblasting with methyl methacrylate (MMA)-based adhesives represents one of the most promising approaches for dental PEEK modification [5,22,27,28]. Since adequate wetting of the PEEK surface is important for achieving intimate contact with adhesives, surface treatments that improve wettability, such as plasma treatment, also enhance the bond strength when combined with MMA adhesives [23,25,26].

Plasma and corona discharge (CD) treatments are widely used to improve surface wettability and adhesion in industrial applications. Plasma treatment employs electrically generated gas plasma to remove surface contaminants and introduce hydrophilic functional groups. In contrast, CD represents a localized electrical discharge that occurs when a gas undergoes electrical breakdown between electrodes under a high voltage and is characterized by a blue glow. Since the gas is ionized into plasma, CD treatment is considered a type of plasma treatment [29,30]. CD treatment can be performed under atmospheric pressure and is widely used not only for surface modification but also for water/air purification and static electricity control [31]. CD treatment has also been employed in the surface modification of commercial PEEK, where it has been reported to improve the bond strength between PEEK films and epoxy adhesive films [8,32]. However, the effects of CD treatment on the adhesion of dental PEEK have yet to be investigated.

Accordingly, the purpose of this study is to evaluate the effects of CD treatment on the bonding of resin cement to mirror-polished (MP) and sandblasted (SB) dental PEEK using an MMA-based adhesive. Tensile bond strength (TBS) and failure modes at the bonding interface are evaluated and compared. Furthermore, to complement the adhesion evaluation, the surface roughness (Ra), surface topography, and contact angles are analyzed to characterize the CD treatment-induced surface modifications. The null hypothesis is that CD treatment does not affect the bonding performance of PEEK to resin cement or the failure modes.

## 2. Materials and Methods

### 2.1. Specimen Preparation

Table 1 lists the materials used in the current study. Specimens for the bonding tests were prepared according to a previously reported method [33], with minor modifications. Specifically, the PEEK CAD/CAM blocks were sliced into disks (12.0 × 14.4 × 3.0 mm), preparing 136 replicates in total (80 for TBS testing, 32 for surface roughness measurements, and 24 for water contact angle measurements). All specimens were ground with 600-grit silicon carbide (SiC) papers under running water, and subsequently randomly divided into two groups (i.e., the MP and SB groups). The MP group was further ground with 1500-grit SiC paper, followed by buff polishing with a 1 μm alumina suspension. The SB group was sandblasted with 50 μm Al_2_O_3_ particles at 0.3 MPa for 15 s at a distance of 10 mm using an air-abrasion unit (Basic eco, Renfert GmbH, Hilzingen, Germany).

### 2.2. Surface Treatment

**Figure 1:**
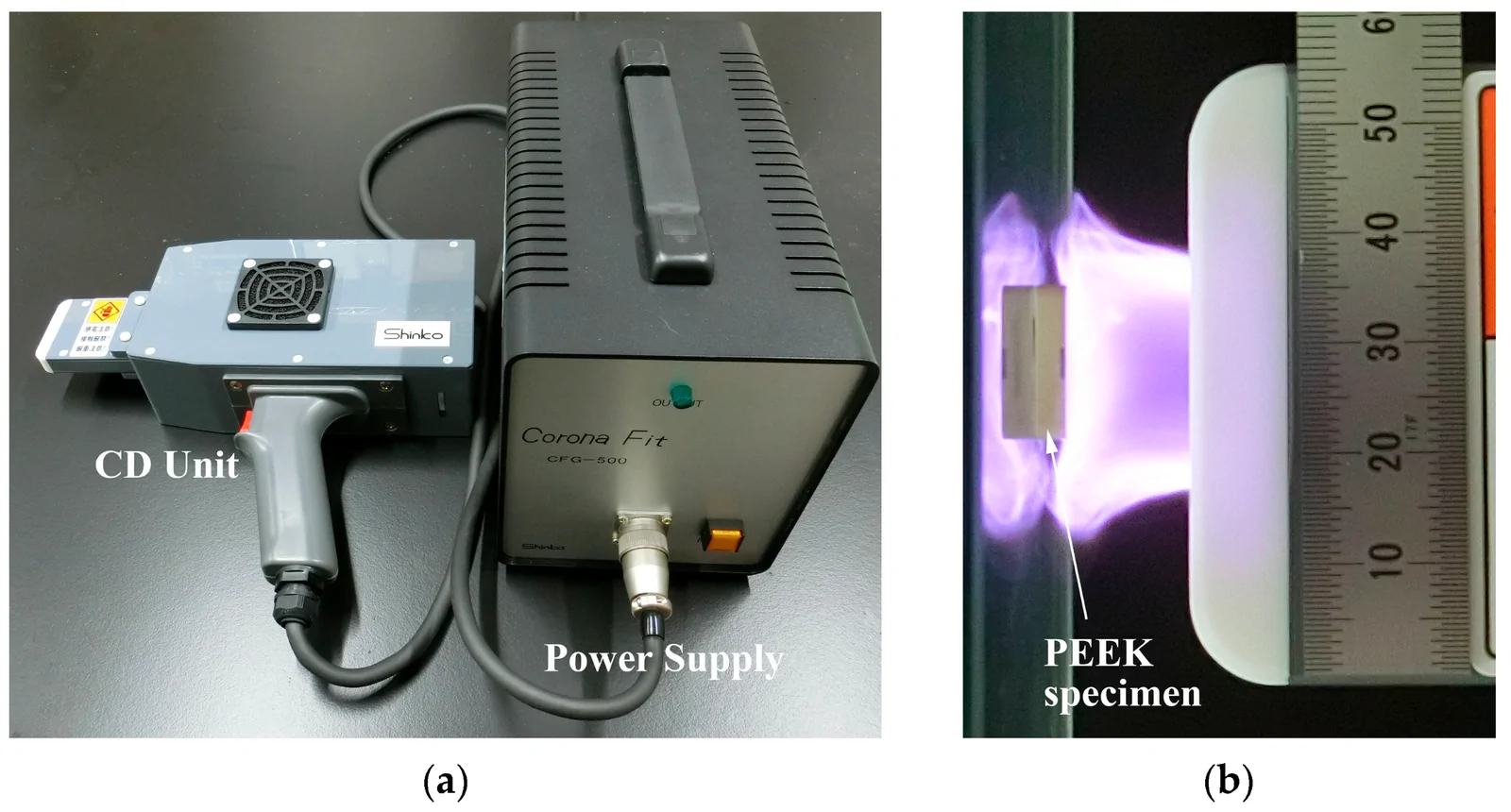
Photographic images of the CD treatment apparatus. (**a**) Overview of the system; (**b**) CD treatment of the PEEK specimen. Abbreviations: CD, corona discharge; PEEK, polyetheretherketone.

The SB and MP specimens were ultrasonically cleaned in distilled water for 10 min, followed by cleaning in 98% ethanol for 5 min and subsequent air drying. Each group was further subdivided into CD-treated [CD(+)] and -untreated [CD(−)] groups, wherein the CD(+) group was subjected to CD treatment for 2 min at a distance of 10 mm in air (CoronaFit CFG-500: frequency = 50 kHz, high-frequency output = 108 W, normal rated power = 360 VA, Shinko Electric & Instrumentation Co., Ltd., Osaka, Japan). The apparatus used for CD treatment is shown in Figure 1.

### 2.3. Surface Roughness, Surface Topography Analysis, and Contact Angle Measurements

PEEK specimens, prepared and assigned to four groups [MP-CD(−), MP-CD(+), SB-CD(−), and SB-CD(+)], were subjected to the respective measurements. Specifically, the Ra values of the MP and SB PEEK surfaces before and after CD treatment were measured using a surface roughness meter (Surfcom 50A, Tokyo Seimitsu Co., Ltd., Tokyo, Japan) (*n* = 8, one position per specimen). Changes in the surface topography of the PEEK surfaces with and without CD treatment were observed using confocal laser scanning microscopy (CLSM; OLS4100, Olympus, Tokyo, Japan). The static water contact angle of the PEEK surface with and without CD treatment was also measured 10 s after dispensing a droplet (1.0 μL) of distilled water onto the surface at 23 °C (DM-501 automatic contact angle meter, Kyowa Interface Science Co., Ltd., Niiza, Japan) (*n* = 6, one position per specimen).

### 2.4. Bonding Procedures

An adhesive was applied to all specimens for 10 s, followed by air-drying and light-curing for 10 s at a distance of 5 mm. For the CD(+) group, to prevent ambient contamination during the transition between procedures, the treated specimens were temporarily stored in a sealed container for ~30 s prior to adhesive application. The bonding area was defined by covering the surface with 0.1 mm-thick double-sided tape (Nicetack strong, Nichiban Co., Ltd., Tokyo, Japan) containing a 4 mm-diameter hole. A stainless-steel rod (8 mm diameter) that had been subjected to sandblasting and priming was bonded to the PEEK specimens using a dual-cure resin cement and light-cured for 10 s from four different directions at a 45° angle (40 s in total). The bonded assemblies were fixed and maintained at 37 °C and 100% relative humidity for 30 min, then followed by storage in distilled water at 37 °C for 24 h.

### 2.5. Thermal Cycling and TBS Measurements

**Figure 2:**
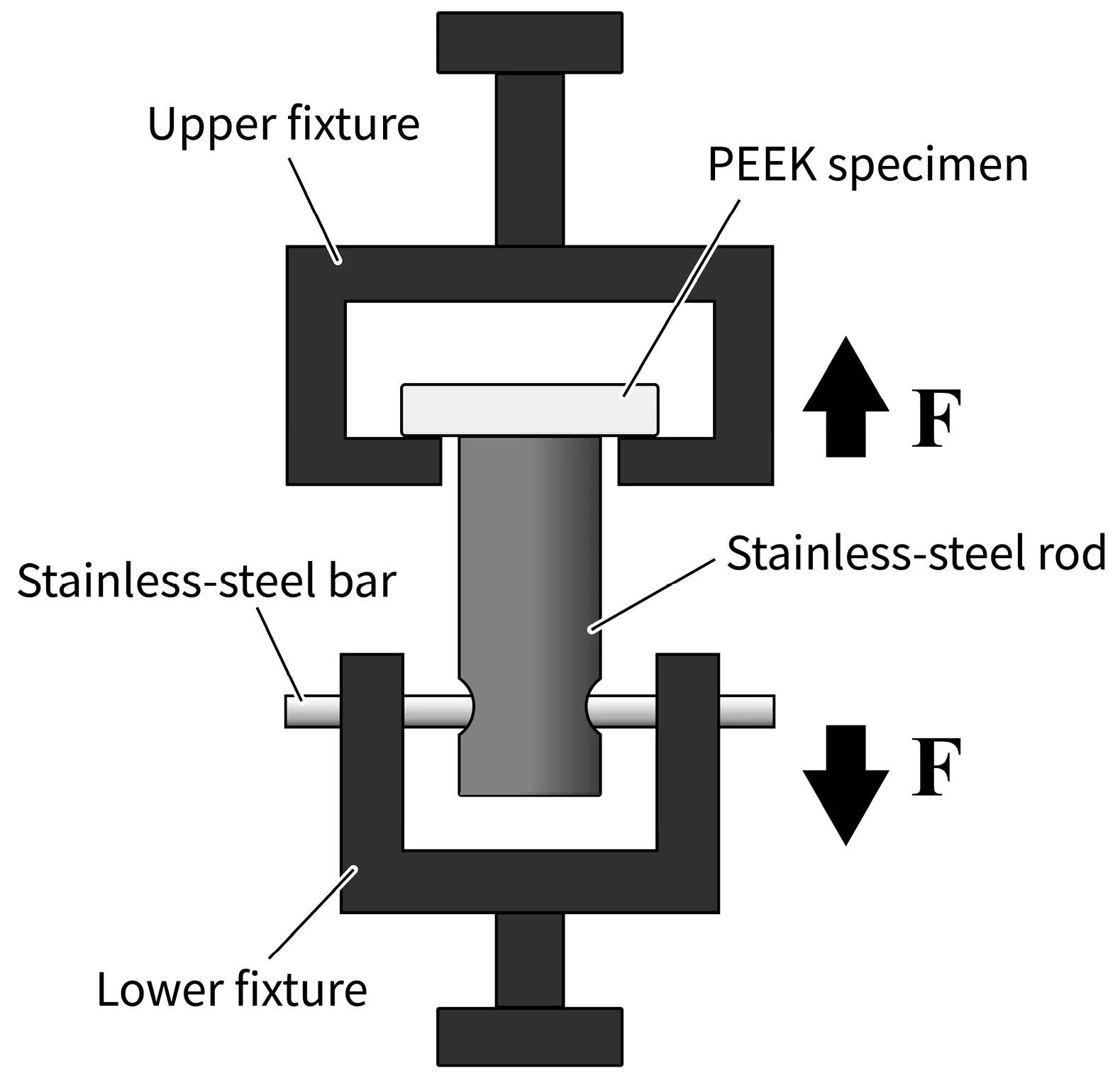
Schematic illustration of TBS testing. Abbreviation: PEEK, polyetheretherketone; TBS, tensile bond strength.

Thermal cycling (TC) was performed under either 0 (TC0) or 5000 cycles (TC5000) using a TC device (K178-08, Tokyo Giken, Setagaya, Japan). The temperature ranged between 5 and 55 °C with a dwell time of 30 s and a transfer time of 5 s per cycle. After cycling, the TBS was measured (EZ-LX universal testing machine, Shimadzu Corp., Kyoto, Japan) at a crosshead speed of 1.0 mm/min (*n* = 10) using the jig shown in Figure 2. The TBS was calculated as the maximum load divided by the bonding area (4 mm diameter).

### 2.6. Failure Mode Analysis

Failure mode analysis of the debonded specimens was performed after TBS testing using scanning electron microscopy (SEM, TM4000Plus, Hitachi High-Tech Corp., Tokyo, Japan) at 20× magnification with an acceleration voltage of 15 kV. The fracture surfaces were classified into four categories: (A) adhesive failure at the PEEK–adhesive interface; (B) mixed failure consisting of adhesive failure and cohesive failure within the resin cement, with <20% of the resin cement remaining on the bonded surface; (C) mixed failure with 20–50% of the resin cement remaining; and (D) mixed failure with >50% of the resin cement remaining.

### 2.7. Statistical Analysis

The data were analyzed using statistical software (JMP version 19, SAS Institute Inc., Cary, NC, USA). The normality and homogeneity of variance were assessed prior to analysis using the Shapiro–Wilk test and Brown–Forsythe tests, respectively. The TBS data did not meet the assumption of homogeneity of variance (Brown–Forsythe, *p* < 0.05). The TBS values (*n* = 10) were analyzed using the Welch one-way analysis of variance (ANOVA) to compare the experimental groups (defined by combinations of initial surface condition, CD treatment, and TC duration), followed by the Welch *t*-test with Bonferroni correction. The *p*-values for the 12 prespecified comparisons were adjusted for multiple comparisons using the Bonferroni correction.

The surface roughness and contact angle data (*n* = 8 and *n* = 6, respectively) did not meet the assumption of homogeneity of variance (Brown–Forsythe, *p* < 0.05) and were transformed using the Box–Cox transformation (λ = 0.5). All inferential statistical analyses were conducted on the Box–Cox-transformed data to satisfy the assumptions of two-way ANOVA (Brown–Forsythe, *p* > 0.05). Descriptive statistics (means and standard deviations) are reported on the original measurement scale to facilitate interpretation. Surface condition and CD treatment were defined as the main factors. When a significant interaction between the surface condition and the CD treatment was detected, simple main effects analyses were performed to evaluate the effect of CD treatment within each surface condition and the effect of surface condition within each CD treatment. The *p*-values for these four prespecified comparisons were adjusted for multiple comparisons using the Bonferroni correction.

To determine the required sample size for the TBS test, a priori power analysis was conducted using G*Power 3.1.9.7 (Heinrich Heine University, Düsseldorf, Germany). The effect size was estimated based on experimental data from the previous study [17] using the same adherend and adhesive system. For this purpose, the parameters were as follows: α error = 0.05, 1 − β error = 0.95, mean differences = 4, standard deviation (SD) = 2 for both groups, effect size (Cohen’s d) = 2, indicating a minimum sample size of 8 specimens per group. A conservative approach was adopted for the sample size determination, and the number of specimens was increased to 10 per group.

Because a priori power analysis was not performed for the surface roughness and contact angle measurements prior to the experiment, a sensitivity power analysis was subsequently conducted to evaluate the detectable effect size for the available sample size. Using G*Power, the analysis was performed for a fixed-effects ANOVA testing the main effects and interactions, with parameters set at α = 0.05, power (1 − β) = 0.80, 4 groups, and a numerator degree of freedom of 1. For total sample sizes of 32 for surface roughness and 24 for contact angle, the analysis indicated that the minimum effect sizes detectable with 80% power were Cohen’s f = 0.513 and 0.601, respectively.

The significance level was set at α = 0.05 for all statistical analyses.

## 3. Results

### 3.1. Surface Roughness Measurements and Surface Topography Analysis

The Ra values of the MP and SB groups before and after CD treatment are listed in Table 2. Two-way ANOVA revealed a significant main effect of the surface condition on Ra (*p* < 0.0004, Cohen’s f = 8.578), as well as a significant interaction between the surface conditions and CD treatment (*p* < 0.0004, Cohen’s f = 1.264). The MP-CD(+) group exhibited a significantly higher Ra value than the MP-CD(−) group (*p* < 0.0004). Conversely, that of the SB-CD(+) group decreased significantly after CD treatment (*p* = 0.0112).

**Figure 3:**
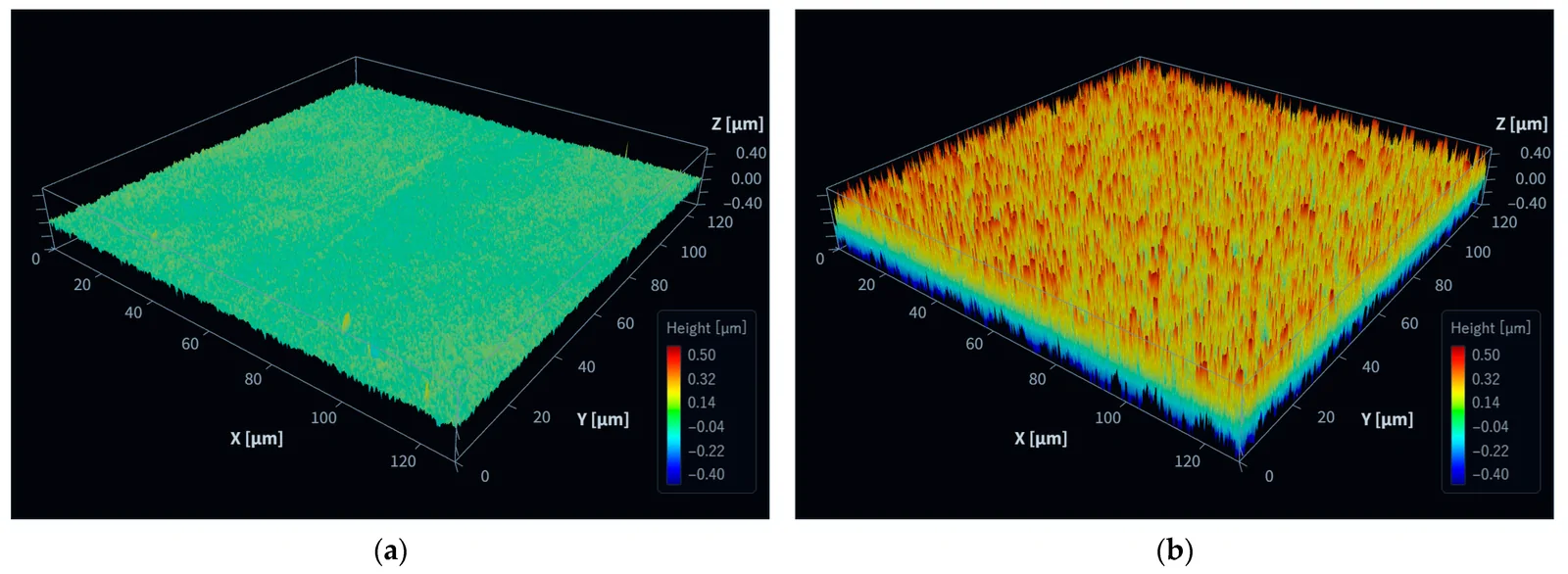
Representative CLSM images of the MP surface before and after CD treatment. Experimental groups: (**a**) MP-CD(−) and (**b**) MP-CD(+). Abbreviations: CLSM, confocal laser scanning microscope; CD, corona discharge; MP, mirror-polished.

Representative 3D CLSM images of MP specimens (Figure 3) show the topographical changes induced by CD treatment. For the MP specimens, CD treatment removed the polishing marks and markedly increased the surface roughness. In contrast, no typical changes in surface topography following CD treatment were observed for the SB specimens.

### 3.2. Contact Angle Measurements

The contact angle values for the PEEK surface with and without CD treatment are listed in Table 3, revealing that the contact angles of both the MP and SB surfaces were significantly reduced after CD treatment. Two-way ANOVA revealed that the surface condition, CD treatment and their interaction with CD treatment significantly affected the contact angle (*p* < 0.0004, Cohen’s f = 1.049, 14.984, and 2.420, respectively). Before CD treatment, the contact angle of the SB group was significantly higher than that of the MP group (*p* < 0.0004), whereas after CD treatment, the contact angle of the SB group was significantly lower than that of the MP group (*p* < 0.0004).

### 3.3. TBS Measurements

**Figure 4:**
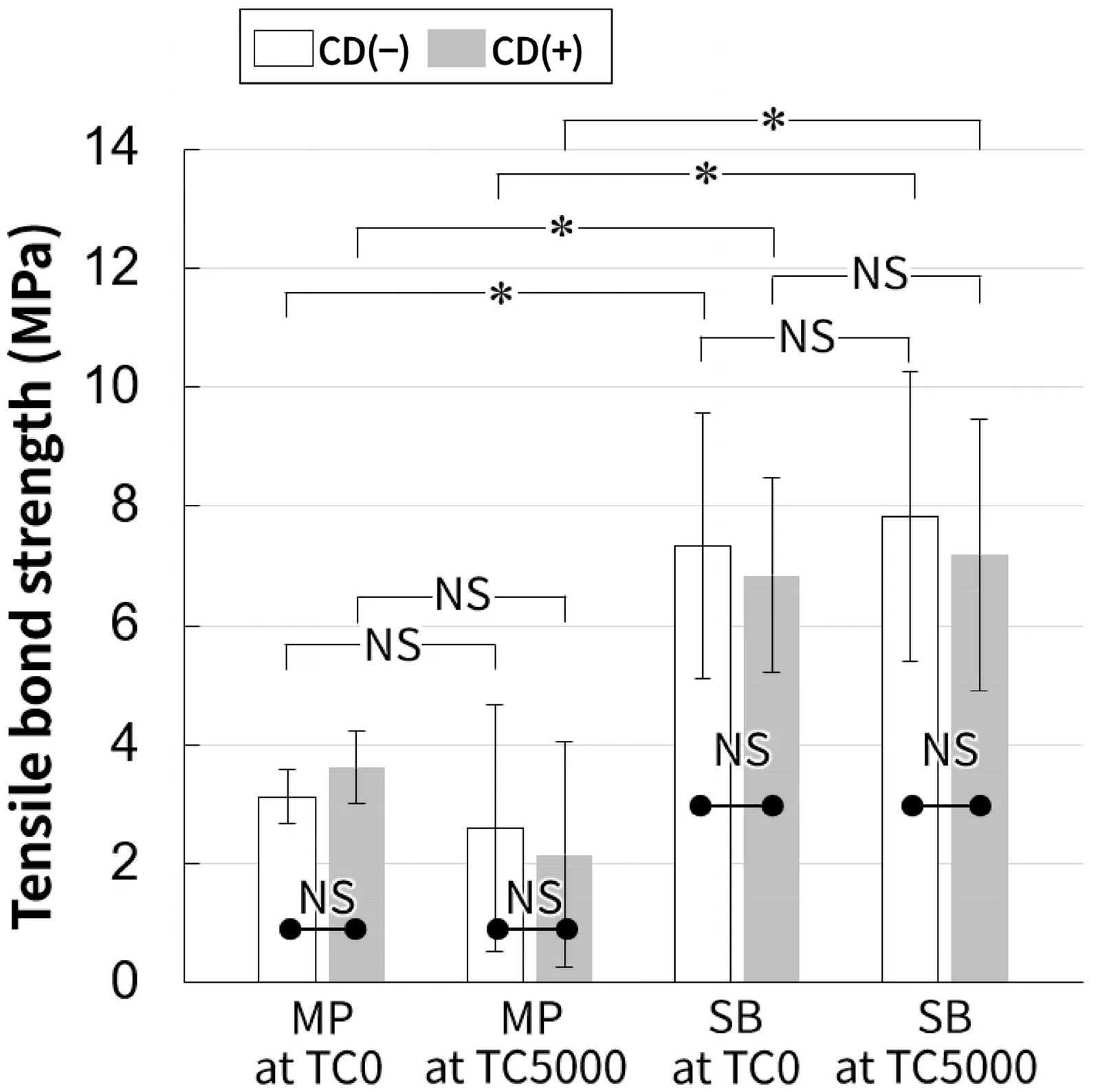
TBS values of different surface conditions with and without CD treatment and under different TC durations (TC0 and TC5000) (*n* = 10). Experimental groups (left to right): MP-CD(−) at TC0; MP-CD(+) at TC0; MP-CD(−) at TC5000; MP-CD(+) at TC5000; SB-CD(−) at TC0; SB-CD(+) at TC0; SB-CD(−) at TC5000; and SB-CD(+) at TC5000. The asterisk indicates a statistically significant difference between the MP and SB groups within each CD treatment condition and each TC duration (Welch *t*-test with Bonferroni correction, *p* < 0.05). Abbreviations: CD, corona discharge; MP, mirror-polished; NS, not significant; SB, sandblasted; TC, thermal cycling.

Figure 4 shows the TBS values for the MP and SB specimens with and without CD treatment and under different TC durations (TC0 and TC5000). Regarding the effect of CD treatment, the MP at TC0, MP at TC5000, SB at TC0, and SB at TC5000 groups exhibited no significant difference between the CD(−) and CD(+) groups (*p* > 0.05). Regarding the effect of surface condition, the SB group exhibited a significantly higher TBS than the MP group, regardless of CD treatment and TC durations (*p* < 0.05). Regarding the effect of TC, no significant difference was observed between the MP at the TC0 and the MP at TC5000 groups, and between the SB at TC0 and the SB at TC5000 groups, regardless of CD treatment (*p* > 0.05). For MP specimens at TC5000, three specimens in each of CD(−) and CD(+) groups could not be measured, and a value of zero was assigned to each specimen for calculation purposes. Exact *p*-values for the above comparisons are presented in Table S1.

### 3.4. Failure Mode Distribution

**Figure 5:**
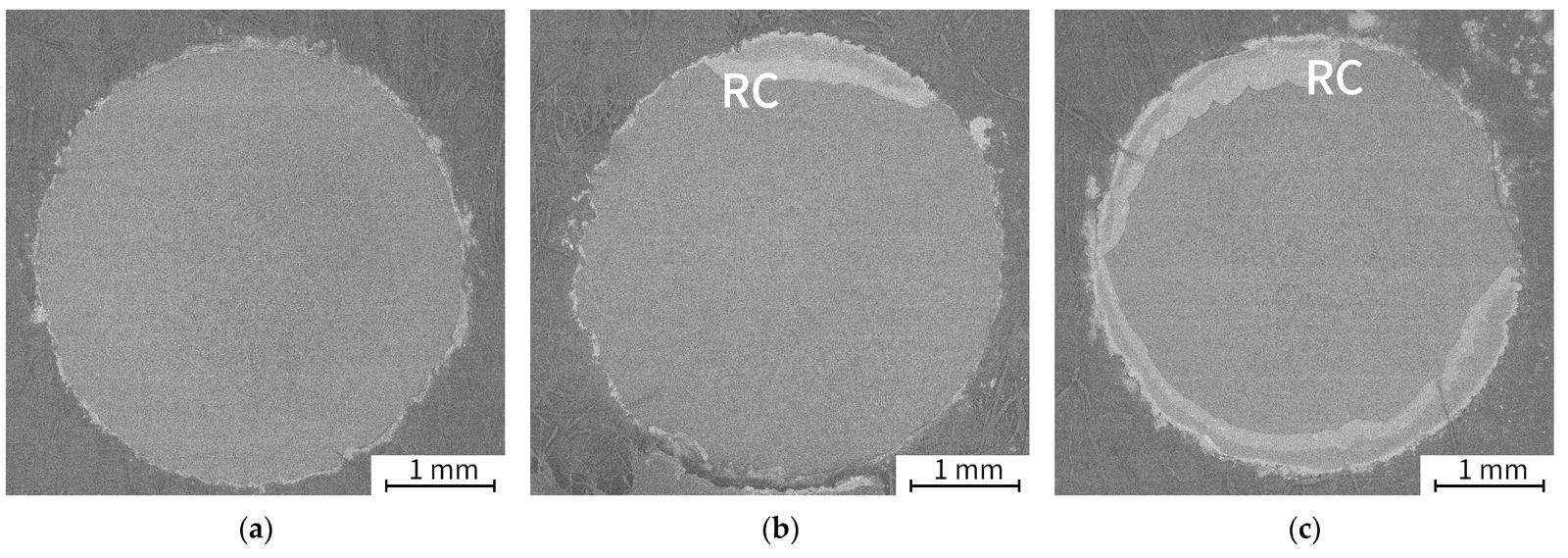
Representative SEM images of the fracture surfaces in the SB group for each failure mode after tensile bond strength testing (20×). (**a**) Adhesive failure at the PEEK–adhesive interface; (**b**) mixed failure with <20% of resin cement remaining on the bonded surface; and (**c**) mixed failure with 20–50% of resin cement remaining on the bonded surface. Abbreviations: RC, resin cement; SEM, scanning electron microscopy; SB, sandblasted.

The distribution of failure modes after TBS testing is summarized in Table 4. Representative SEM images of the fracture surfaces in the SB group for each failure mode are shown in Figure 5, revealing that cohesive failure within the resin cement was more frequently observed in the SB at TC5000 group than in the SB at TC0 group. Comparing the CD(−) and CD(+) groups revealed no difference in the distribution of failure modes in either the SB at TC0 or SB at TC5000 group. All MP specimens exhibited 100% adhesive failure at the PEEK–adhesive interface, regardless of CD treatment.

## 4. Discussion

Due to its biocompatibility and excellent mechanical and chemical properties, PEEK is increasingly being applied as a metal-free dental material. However, due to its lack of polar surface functional groups, conventional bonding systems using functional monomers fail to provide sufficient bond strength [5,6,34]. In clinical practice, sandblasting is commonly employed to enhance mechanical interlocking; thus, sandblasting at 0.3 MPa was used in the current study [6].

Additionally, plasma treatment is a surface modification technology frequently employed to achieve enhanced bonding to polymeric materials [35,36]. In both low-pressure plasma and atmospheric-pressure plasma treatments, plasma is generated by electrical discharge between electrodes. The resulting active species clean the surface, introduce functional groups, and improve both wettability and bonding performance [35,36]. The effect of plasma treatment differs depending on the type of gas introduced, with inert gases such as Ar resulting only in surface cleaning and molecular alteration. In contrast, oxygen incorporates hydrophilic functional groups, including carboxyl and hydroxyl groups [35,36,37].

During CD, surrounding gas molecules are ionized into plasma by electrical discharge, causing charged particles to move along the electric field. Since these excited species emit visible and UV light, the specimen surface can be simultaneously exposed to both charged particles and UV light [30]. CD treatment is typically performed in an atmospheric environment wherein the generated oxygen radicals and ozone remove organic contaminants and introduce surface functional groups [32,36,38]. Consequently, a simple and continuous processing workflow can be achieved without the requirement for vacuum chambers or evacuation systems [29,31,38]. Regarding the aging of CD-treated surfaces, a significant decrease in contact angle has been reported for LDPE within a few days after treatment [39]. In the present study, bonding was achieved within 30 s of treatment, suggesting that degradation caused by aging was negligible. As shown in Figure 1, the CD treatment device is relatively compact, and its application immediately before bonding may minimize aging-induced degradation, rendering it a promising method for clinical applications.

Anticipating these practical and surface modification benefits, the current study investigated the effects of CD treatment on the adhesion properties of dental PEEK. Specifically, the samples were subjected to SB or MP pretreatment, with and without CD treatment under different TC durations prior to the TBS test and failure mode analysis. The MP specimens served as a comparison with the SB specimens to isolate the impact of CD treatment from mechanical interlocking. As complementing measurements, surface roughness (Ra), surface topography, and contact angle were analyzed to characterize the CD treatment-induced surface modifications that could affect adhesion.

The Ra of the MP group increased significantly following CD treatment, as supported by CLSM analysis, whereas that of the SB group decreased slightly. As reported previously [40], CD generates strong filamentary arcs that produce micropinholes on flat substrates, and a similar effect has been reported for low-density polyethylene (LDPE) [38]. Similarly, the changes in Ra observed on the SB surfaces in this study were likely due to the convex regions of the rough SB surface undergoing local melting or ablation caused by the high thermal energy of the corona arc. Thus, while the surface-roughening effect of CD in the MP group at TC0 may have contributed to a slight improvement in bond strength, no such mechanical contribution would be expected in the SB group.

The water contact angles on both the MP and SB surfaces were significantly reduced after CD treatment, suggesting that CD treatment improves the wettability of dental PEEK. Similarly, Popelka et al. [38] reported an improved wettability and increased Ra following CD treatment of a smooth LDPE surface, which is consistent with the current findings. Although low-pressure plasma and atmospheric-pressure plasma treatments have been reported to enhance PEEK surface wettability [10,15,18,20,21,22,41], these plasma treatments do not necessarily increase Ra. Indeed, some studies have shown that low-pressure plasma did not alter the Ra value of PEEK despite improving its wettability [20,22,41], whereas others have found that plasma treatment improved the wettability while increasing the Ra of sandblasted PEEK surfaces [15,18].

Regarding the TBS, no statistically significant improvement was observed following CD treatment in either the MP or SB groups. Nevertheless, within the MP at TC0 group, the CD(+) specimens exhibited a slightly higher mean TBS than the CD(−) specimens, likely due to the increased surface roughness indicated by the Ra values (Table 2) and the surface topography of the MP specimens (Figure 3) following CD treatment. In contrast, for the SB groups, CD treatment slightly reduced the Ra values and the convex regions of the SB surface, thereby accounting for the lack of TBS enhancement at either TC0 or TC5000.

All specimens in the MP groups exhibited adhesive failure at the PEEK–cement interface regardless of CD treatment and TC. In the SB groups, regardless of CD treatments, both adhesive failure (A) and mixed failure (B) were observed in roughly equal proportions at TC0; however, at TC5000, mixed failure (B) dominated. This could be attributed to the roughened surface of the SB at TC5000 specimens, which may have enhanced interfacial bonding, combined with further polymerization of resin cement during the TC process. Consequently, the proportion of adhesive failure may decrease, while that of mixed failure may increase. Based on these findings that TBS and the failure mode did not change following CD treatment, the null hypothesis could not be rejected.

In the present study, a TC protocol of 5000 cycles was employed to simulate hydrothermal aging. Gale and Darvell suggested that 10,000 thermal cycles represent one clinical service year [42]; therefore, 5000 cycles correspond to half a year of clinical service, which is considered sufficient for estimating initial bonding durability. However, it should be noted that the 5000 TC may have been insufficient to produce a significant change in the TBS and the failure modes of tested PEEK. Further investigations should therefore evaluate the effects of longer thermocycling, such as 10,000 cycles, as employed by Lai et al. [26] to assess changes following surface treatments.

Regarding previous attempts to improve the adhesion of dental PEEK, several studies have reported the positive effect of plasma treatment. For instance, SiC-polished and sandblasted PEEK surfaces treated with low-pressure plasma [18,22,23] or atmospheric-pressure plasma [25,26] exhibited significantly improved shear bond strengths (SBS) when combined with dental adhesive systems. However, Ito et al. [24] evaluated the effect of low-pressure plasma treatment on the TBS between PEEK and resin cement and reported no significant change in bond strength, which is consistent with the present findings. While no previous studies have subjected dental PEEK to CD treatment, studies performed on a commercial-grade PEEK film revealed that CD treatment increases the lap shear strength [32] and improves the peel force [8] upon bonding to industrial epoxy adhesives.

The majority of previous studies have concluded that plasma or CD treatment enhances both bond strength and wettability; however, such an effect was not observed in the present study. This discrepancy may be attributed to several factors. First, most previous studies evaluated adhesion based on SBS testing [15,18,21,22,23,25,26,28,41], while TBS testing, as employed in the present study, has been used in some previous studies [23,33,43,44]. Additionally, Stawarczyk et al. [27] noted that SBS reflects not only the bond strength but also the overall stability of the bonded interface. Therefore, the difference in testing methodology may account for the discrepancy. Second, the experimental parameters, including the discharge output, gas type, treatment duration, and aging protocols, varied widely among previous studies [5,13]. These findings indicate that the bond strength of PEEK can differ depending on the specific experimental parameters of surface treatment and testing methods.

Regarding the limitations of this study, it is noted that different CD treatment conditions and testing methods were not compared. Additionally, the surface properties were evaluated only based on water contact angle measurements, and no chemical analysis was performed. Regarding the bonding test, TBS was employed without comparison with SBS. Furthermore, TC protocols exceeding 5000 cycles were not evaluated, and different bonding systems were not compared. Thus, future studies should focus on evaluating changes in the surface chemical properties induced by CD treatment and systematically optimizing the CD parameters in combination with sandblasting. Further comparisons of bonding strength testing methods, longer TC protocols, and different bonding systems are also warranted to establish reliable adhesion of PEEK.

In conclusion, although CD treatment of dental PEEK significantly improved the surface wettability and altered the surface roughness depending on the initial surface condition, these superficial modifications were insufficient to produce a measurable enhancement of the TBS to resin cement. These findings suggest that for dental PEEK bonding with MMA-based adhesive systems, the mechanical interlocking provided by sandblasting remains the predominant contributor to bond strength, whereas the chemical and physical alterations induced by this specific CD protocol appear to have a limited effect.

## Figures and Tables

**Table 1:** Materials used in the current study.

Material *	Product Name	Composition	Application Procedure
PEEK	VESTAKEEP^®^ DC4450G	Polyetheretherketone, TiO_2_, others	−
Primer	M.L Primer	10-MDDT, 6-MHPA, acetone	Apply the primer onto the bottom of a stainless-steel rod for 10 s, air-dry.
Adhesive	CAD/CAM Resin Adhesive	MMA, UDMA, acetone, camphor-quinone, reducing agent, polymerization initiator, others	Apply the adhesive onto the PEEK surface for 10 s, air-dry and light cure for 10 s.
Cement	ResiCem EX	S-RPG filler, Bis-GMA, silicate glass, initiators, others	Employ a dual-barrel syringe to mix pastes A and B upon dispensing, and light cure for 40 s.

* Manufacturer: PEEK, Daicel-Evonik Co., Tokyo, Japan; primer, adhesive, and cement, Shofu Inc., Kyoto, Japan. Abbreviations: 6-MHPA, 6-methacryloyloxyhexyl phosphonoacetate; 10-MDDT, 10-methacryloyloxydecyl 6,8-dithiooctanoate; Bis-GMA, bisphenol A glycidyl-methacrylate; CAD/CAM, computer-aided design and computer-aided manufacturing; MMA, methyl methacrylate; S-RPG filler, surface pre-reacted glass-ionomer filler; UDMA, urethane dimethacrylate.

**Table 2:** Surface roughness (Ra) values of different surface conditions before and after CD treatment (*n* = 8).

CD Treatment	MP (µm)	SB (µm)
CD(−)	0.034 ± 0.013 ^Aa^	0.575 ± 0.043 ^Ab^
CD(+)	0.078 ± 0.009 ^Ba^	0.501 ± 0.059 ^Bb^

Values are presented as the mean ± SD. Experimental groups: MP-CD(−); MP-CD(+); SB-CD(−); and SB-CD(+). Different uppercase letters indicate statistically significant differences between CD(−) and CD(+) within the same column (simple main effects analyses with Bonferroni correction, Bonferroni-adjusted *p* < 0.05). Different lowercase letters indicate statistically significant differences between MP and SB within the same row (simple main effects analyses with Bonferroni correction, Bonferroni-adjusted *p* < 0.05). Abbreviations: CD, corona discharge; MP, mirror-polished; SB, sandblasted.

**Table 3:** Contact angles for the different surface conditions with/without CD treatment (*n* = 6).

CD Treatment	MP (Degree)	SB (Degree)
CD(−)	78.4 ± 6.5 ^Aa^	112.8 ± 3.5 ^Ab^
CD(+)	6.0 ± 0.4 ^Ba^	3.1 ± 1.1 ^Bb^

Values are presented as the mean ± SD. Experimental groups: MP-CD(−); MP-CD(+); SB-CD(−); and SB-CD(+). Different uppercase letters indicate statistically significant differences between CD(−) and CD(+) within the same column (simple main effects analyses with Bonferroni correction, Bonferroni-adjusted *p* < 0.05). Different lowercase letters indicate statistically significant differences between MP and SB within the same row (simple main effects analyses with Bonferroni correction, Bonferroni-adjusted *p* < 0.05). Abbreviations: CD, corona discharge; MP, mirror-polished; SB, sandblasted.

**Table 4:** Distribution of the failure modes after tensile bond strength testing.

CD Treatment	MP at TC0	MP at TC5000	SB at TC0	SB at TC5000
CD(−)	10/0/0/0	10/0/0/0	4/6/0/0	0/8/2/0
CD(+)	10/0/0/0	10/0/0/0	5/5/0/0	1/9/0/0

Values are presented as the number of specimens for each failure mode (A/B/C/D). A: Adhesive failure at the PEEK–adhesive interface; B: mixed failure with <20% of resin cement remaining on the bonded surface; C: mixed failure with 20–50% of resin cement remaining on the bonded surface; and D: mixed failure with >50% of resin cement remaining on the bonded surface. Experimental groups: MP-CD(−) at TC0; MP-CD(+) at TC0; MP-CD(−) at TC5000; MP-CD(+) at TC5000; SB-CD(−) at TC0; SB-CD(+) at TC0; SB-CD(−) at TC5000; and SB-CD(+) at TC5000. Abbreviations: CD, corona discharge; MP, mirror-polished; SB, sandblasted; TC, thermal cycling.

## Data Availability

The data presented in this study are available upon request from the corresponding author.

## Supplementary Materials

The following supporting information can be downloaded at https://www.mdpi.com/article/10.3390/dj14090609/s1, Table S1: Exact *p*-values for comparisons among the experimental groups for TBS values.

## Abbreviations

The following abbreviations are used in this manuscript:

PEEK	Polyetheretherketone
CD	Corona discharge
CD(+)	CD-treated
CD(−)	CD-untreated
SiC	Silicon carbide
MP	Mirror-polished
SB	Sandblasted
TBS	Tensile bond strength
TC	Thermal cycling
CLSM	Confocal laser scanning microscopy
SEM	Scanning electron microscopy
SBS	Shear bond strength
