# Supplementary material for: Effect of Corona Discharge Treatment on the Adhesion of Polyetheretherketone Using Resin Cement
Source: Dent J (Basel). 2026 Sep 20;14(9):609. doi: 10.3390/dj14090609 (PMC13605592; doi:10.3390/dj14090609)
Supplement: Supplementary file 1 [file dentistry-14-00609-s001.zip › Table S1.pdf]

Supplementary Materials

The exact *p*-values for comparisons among the experimental groups for the TBS values reported in section 3.3. (TBS Measurements) are provided in Table S1.

Table S1. Exact *p*-values for comparisons among the experimental groups for TBS values

Comparisons among Experimental Groups	*P* value
**The effect of CD treatment**	
MP-CD(−) at TC0 vs MP-CD(+) at TC0	$p = 1.000$
MP-CD(−) at TC5000 vs MP-CD(+) at TC5000	$p = 1.000$
SB-CD(−) at TC0 vs SB-CD(+) at TC0	$p = 1.000$
SB-CD(−) at TC5000 vs SB-CD(+) at TC5000	$p = 1.000$
**The effect of surface condition**	
MP-CD(−) at TC0 vs SB-CD(−) at TC0	$p = 0.002$
MP-CD(−) at TC5000 vs SB-CD(−) at TC5000	$p = 0.001$
MP-CD(+) at TC0 vs SB-CD(+) at TC0	$p = 0.001$
MP-CD(+) at TC5000 vs SB-CD(+) at TC5000	$p = 0.001$
**The effect of TC**	
MP-CD(−) at TC0 vs MP-CD(−) at TC5000	$p = 1.000$
MP-CD(+) at TC 0 vs MP-CD(+) at TC5000	$p = 0.475$
SB-CD(−) at TC0 vs SB-CD(−) at TC5000	$p = 1.000$
SB-CD(+) at TC0 vs SB-CD(+) at TC5000	$p = 1.000$
